# Manual and Fully Automated Chemotaxis-Based Cancer Screening Yield Equivalent Performance: A Nine-Month Real-World, Side-by-Side Study of the N-NOSE Workflow

**DOI:** 10.3390/biomedicines14071567

**Published:** 2026-07-13

**Authors:** Hideyuki Hatakeyama, Masayo Morishita, Hirotaka Oshida, Takaaki Hirotsu, Eric di Luccio

**Affiliations:** HIROTSU BIO SCIENCE Inc., 22F The New Otani Garden Court, 4-1 Kioi-cho, Chiyoda-ku, Tokyo 102-0094, Japan; h.hatakeyama@hbio.jp (H.H.); m.morishita@hbio.jp (M.M.); h.oshida@hbio.jp (H.O.); hirotsu@hbio.jp (T.H.)

**Keywords:** N-NOSE, *Caenorhabditis elegans*, chemotaxis assay, multi-cancer early detection, automated analysis, CSA, quality control, analytical equivalence, reproducibility, urine biomarker

## Abstract

**Background/Objectives:** The N-NOSE test is a non-invasive, urine-based multi-cancer screening assay that uses *Caenorhabditis elegans* chemotaxis toward cancer-associated volatile organic compounds in human urine. Scaling the test from a manual research-grade workflow to a high-throughput clinical service has required automation, and the central question this raises, centering around whether mechanization alters the analytical performance of the test, must be answered with operational, not bench-top, data. **Methods:** Here, we present a nine-month (January–September 2023) real-world, side-by-side comparison of the two workflows operating under their actual routine clinical laboratory conditions: the manual chemotaxis assay performed by trained technicians at the Fukuoka Research and Development Center (R&D) and the fully automated Chemotaxis Scoring Apparatus (CSA) running continuously at the Tokyo Testing Center. **Results:** The manual workflow generated 551 paired chemotaxis index (CI) measurements from positive-control (PC) and negative-control (NC) synthetic urine/volatile organic compound (VOC)-mimic reference materials at each of two standard urine dilutions (10^−1^ and 10^−2^); over the same period, the CSA processed 2448 quality control samples (612 per control type) with both biobank-derived urine-based comparison materials and synthetic volatile organic compound reference standards. Both workflows produced large, highly significant, and quantitatively comparable PC-versus-NC separation under genuine operating conditions (manual: Δ_CI = 0.096 and 0.103; Welch’s *t* = 19.83 and 21.95; *p* < 0.0001; Cohen’s *d* = 1.19 and 1.32; CSA risk scale Δ_P–*N* = 14.47 with biobank-derived urine-based materials and 10.17 with synthetic VOC standards). The CSA risk score is a linear, monotonic transformation of the CI. Standardized separation is directly comparable across workflows and is concordant (Cohen’s *d*: manual 1.19–1.32; CSA 0.80–1.44, all large); the manual and automated processes therefore show no meaningful difference in discriminative performance. Because the CSA mechanizes only the handling of worms, samples, and machine-vision counting around an unchanged biological transducer, the live nematode, analytical equivalence is the predicted outcome, and these data confirm it at scale in a real clinical laboratory setting. **Conclusions:** Automation of the N-NOSE process does not compromise its ability to discriminate cancer from non-cancer urine. These results provide real-world evidence supporting the validity, reproducibility, and reliability of the N-NOSE testing process and large-scale validation studies.

## 1. Introduction

*Caenorhabditis elegans* possesses one of the most sophisticated olfactory systems in the animal kingdom—roughly 1200 candidate chemoreceptor genes, about three times the number found in humans, are deployed across only ~302 neurons, of which 32 are dedicated chemosensory neurons [1,2]. Detection of volatile organic compounds (VOCs) is mediated primarily by the AWA and AWC amphid neurons through G-protein-coupled receptor (GPCR) signaling cascades [3,4], and odor-receptor matching studies have shown that distinct receptor subsets are recruited depending on odorant concentration and class [5]. This biology underpins wild-type *C. elegans*’s ability to detect trace levels of cancer-associated VOCs in urine [6].

The molecular target of N-NOSE is not a single biomarker but a composite signature of cancer-associated urinary VOCs—spanning chemical classes such as organic acids, aldehydes, ketones, alcohols, and aromatic and phenolic compounds—recognized in combination rather than individually [7,8]; distinct olfactory receptor subsets are recruited according to odorant class and concentration [5], so this deliberately multiplexed, pan-VOC readout, rather than reliance on any single metabolite, is what enables one test to flag a broad range of cancer types [6,8]. Leveraging this innate chemosensory capacity, Hirotsu and colleagues developed the N-NOSE test, a non-invasive multi-cancer screen that scores the chemotactic response of wild-type *C. elegans* toward 100×- and 10×-diluted human urine [6]. Subsequent clinical and real-world studies have reported sensitivities and specificities consistent with the original report and extended the test’s applicability across multiple cancer types and clinical contexts, including gastrointestinal [9,10], pancreatic [11,12], breast, esophageal, and hematological malignancies [13,14,15,16], as well as to post-operative surveillance [13,14,15,16,17]. Independent academic groups outside Japan have also validated the basic chemotactic discrimination principle [7,18], and a recent multi-institutional real-world dataset confirmed robust performance in routine clinical use [19,20].

Since 2020, translating N-NOSE from a research-grade manual assay into a routine clinical service in Japan has required scaling throughput by orders of magnitude while preserving the integrity of the underlying biological measurement. To meet this need, we developed the Chemotaxis Scoring Apparatus (CSA), a custom-built, fully automated platform that mechanically replicates each step of the manual N-NOSE inspection process, nematode recovery, dispensing, temperature-controlled incubation, and worm-position counting, under identical conditions. Conceptually, the CSA automates only the preparatory and readout handling around the assay; the biological transducer (the living, wild-type nematode) and the detection principle (chemotaxis toward cancer-associated VOCs) are unchanged. As such, manual and automated analyses are expected to yield equivalent separation of cancer from non-cancer urine, but this expectation has not been documented in a peer-reviewed forum.

Here, we fill that gap through a nine-month (January–September 2023) real-world, side-by-side comparison of the two workflows operating in parallel under their actual routine clinical laboratory conditions: manual analysis by trained technicians at the Fukuoka R&D Center and fully automated analysis on the CSA at the Tokyo Testing Center. We compare the two workflows at the level of the raw chemotaxis index (CI), the assay’s immediate biological readout, rather than at the level of downstream risk scale conversion, and we present complementary CSA quality control data acquired with both biobank-derived urine-based comparison materials and synthetic VOC reference standards. The objective is to provide a concise analytical-equivalence reference based on real operational data rather than bench-top experiments that supports the validity, reproducibility, and reliability of the N-NOSE testing process and large-scale validation work.

## 2. Materials and Methods

### 2.1. Study Design and Sample Period

We performed a retrospective analysis of prospectively collected quality control (QC) data generated during routine N-NOSE testing operations from 1 January 2023 to 30 September 2023. Two parallel workflows were evaluated: (i) manual chemotaxis analysis performed by trained technicians at the Fukuoka R&D Center (HIROTSU BIO SCIENCE Inc., Fukuoka, Japan) and (ii) automated chemotaxis analysis performed by the CSA platform at the Tokyo Testing Center (HIROTSU BIO SCIENCE Inc., Tokyo, Japan). Both sites processed paired positive-control (PC) and negative-control (NC) synthetic urine/VOC-mimic reference materials on every operational day, in parallel with clinical test specimens. The PC and NC routine QC reference materials were synthetic urine/VOC mimics prepared in situ as cocktails of volatile organic compounds formulated to reproduce the expected positive- and negative-control chemotactic responses; these PC/NC reference materials were not of human origin. When authentic human urine was used for R&D/QC comparison, it consisted solely of pre-existing, de-identified biobank specimens purchased for R&D use under the provider’s authorization/terms and supplied without donor-identifying information or patient-level clinical data. The reference materials and any biobank-derived comparison materials were stored as frozen aliquots at −20 °C and were thawed, diluted, and assayed identically to clinical specimens on each operational day (batch), with parent and diluted aliquots retained for at least 100 days. A control passes QC only if its CI falls within pre-established acceptance ranges at both standard dilutions (net attraction for PC; near-zero or net avoidance for NC); a control that behaves outside these validated expectations triggers an automated batch flag and retesting. The exact numerical acceptance thresholds and the risk scale algorithm are proprietary to HIROTSU BIO SCIENCE Inc., and are not disclosed. Only de-identified QC data are reported here; no patient-level clinical information was analyzed. No new human participants were recruited, no intervention was performed, and no new human specimens were collected for this retrospective analysis.

### 2.2. Manual Chemotaxis Assay (Fukuoka R&D Center)

The manual N-NOSE chemotaxis assay was performed as established previously [6,9] and follows the classical *C. elegans* chemotaxis protocol of Ward [21]. Wild-type *C. elegans* (Bristol N2) were synchronized, cultivated on Nematode Growth Medium (NGM) plates seeded with *Escherichia coli* NA22, and harvested at the adult stage, holding eggs arranged in two rows. Reference samples, including PC/NC synthetic urine/VOC-mimic materials and, where applicable, de-identified biobank-derived urine materials, were diluted 1:10 (10^−1^) and 1:100 (10^−2^) in assay buffer. Dilution in a defined buffer is an intrinsic normalization step: it attenuates inter- and intra-individual variation in overall urine concentration (e.g., from hydration status, diet, or time of day) and lowers the high osmolarity, urea, and salt load of neat urine that would otherwise provoke non-specific osmotic avoidance and confound the chemotactic readout. Testing every sample at two fixed dilutions additionally provides an internal dose-response consistency check, so classification does not rest on any single concentration point; neat (undiluted) urine is therefore not used. For routine N-NOSE testing, urine is collected under a standardized pre-analytical protocol (fasting first-morning midstream void where feasible, frozen at −20 °C within approximately 2 h of collection), which further constrains these pre-analytical sources of variability [6,17]. Approximately 50–100 nematodes were dispensed onto the center of a 9 cm assay plate marked with diametrically opposed sample and control spots and incubated for 30 min at 23 °C. After incubation, the whole view of the assay plate was photographed, and simultaneously, the number of worms in the sample (N_S), control (N_C), and origin areas was automatically counted under an XG-X imaging unit (KEYENCE, Tokyo, Japan) (machine-vision counting). The chemotaxis index was calculated as CI = (N_S − N_C)/(N_S + N_C). The CI is a normalized preference score bounded between −1 and +1: a value of +1 indicates that all scored nematodes migrated to the sample (urine) spot (maximal attraction), −1 indicates that all migrated to the opposite control spot (maximal avoidance), and 0 indicates that there is no net preference (equal distribution of worms). Positive CI values thus denote net attraction toward the tested urine, and negative values denote net avoidance of the tested urine; the index follows the classical chemotaxis index formulation of Ward [21]. For each operational day, paired PC and NC controls were assayed in parallel at both dilutions. Over the nine-month study period, this yielded *N* = 551 PC and *N* = 551 NC measurements at each of the two dilutions.

### 2.3. Automated Chemotaxis Assay (CSA; Tokyo Testing Center)

**Figure 1:**
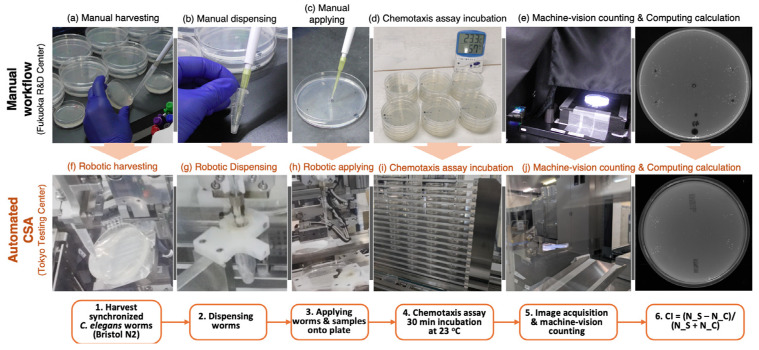
The Chemotaxis Scoring Apparatus (CSA) faithfully replicates every step of the established manual N-NOSE inspection workflow. Each step of the manual N-NOSE inspection workflow (top row, (**a**–**e**), Fukuoka R&D Center) and the corresponding fully automated modules of the CSA (bottom row, (**f**–**j**), Tokyo Testing Center) are displayed. Reagents, plate geometry, incubation time and temperature, and CI calculation are identical between the manual and automated workflows; only sample handling of each step is mechanized. All photographs were acquired in situ within the routine N-NOSE testing environment.

The Chemotaxis Scoring Apparatus (CSA) is a proprietary, fully automated platform designed to replicate the manual N-NOSE inspection process at high throughput (Figure 1a–j). The CSA integrates (i) automated recovery of cultured nematodes from culture plates; (ii) robotic dispensing of a calibrated nematode suspension onto chemotaxis assay plates via precision liquid handling; (iii) temperature-controlled (23 °C) chemotaxis incubation; and (iv) machine-vision-based automated counting of worms in the sample, control, and origin zones. The assay reagents, plate geometry, incubation time and temperature, and CI calculation are identical to those used in the manual workflow. During the same January–September 2023 period, the CSA processed 2448 QC samples (612 per control type, split between biobank-derived urine-based comparison materials and synthetic VOC reference standards). For routine clinical reporting, CSA outputs are post-processed into a proprietary risk scale score; the present analysis emphasizes the raw CI-level data to allow for a direct manual-versus-automated comparison, with CSA risk scale QC reported as a complementary stability dataset.

### 2.4. Statistical Analysis

Descriptive statistics (*N*, mean, standard deviation [SD], standard error of the mean [SEM], median, minimum, maximum) were computed for PC and NC CI distributions at each dilution. Differences between PC and NC distributions were assessed using two-sided Welch’s *t*-tests (unequal-variance) and confirmed by non-parametric Mann-Whitney U tests, given mild departures from normality on Shapiro-Wilk testing in the larger samples. Effect sizes were quantified by Cohen’s d using the pooled standard deviation. Equality of group variances was assessed by Levene’s test. The PC–NC separation was summarized by Δ = mean(PC)–mean(NC). All analyses were performed in Python v3.11 https://www.python.org/downloads/release/python-3110/ (accessed on 1 May 2026) using NumPy v1.26 https://numpy.org/doc/stable/release/1.26.0-notes.html (accessed on 1 May 2026) and SciPy v1.13 https://docs.scipy.org/doc/scipy-1.13.0/ (accessed on 1 May 2026). A two-sided *p*-value < 0.05 was considered statistically significant. The interpretation framework for quality control verification follows general Clinical & Laboratory Standards Institute (CLSI) guidance for user verification of precision.

## 3. Results

### 3.1. Manual Analysis Yields Large, Robust PC–NC Separation at the CI Level Under Real-World Operating Conditions

**Figure 2:**
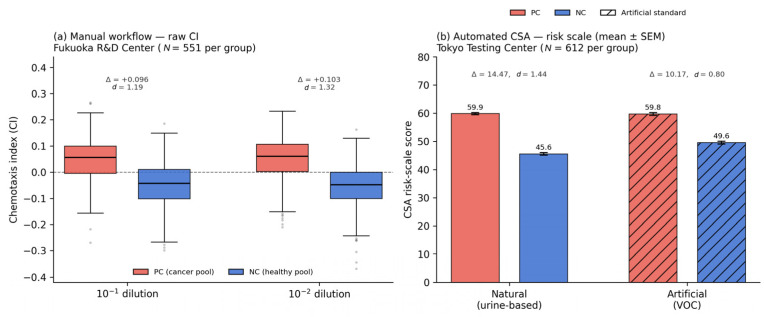
Quality control performance of the manual and automated N-NOSE workflows over the same nine-month period (January–September 2023). (**a**) Box-and-whisker plots of raw chemotaxis index (CI) values for positive controls (PC; red) and negative controls (NC; blue) at 10^−1^ and 10^−2^ urine dilutions, manual analysis at the Fukuoka R&D Center (*N* = 551 per group). Boxes show the interquartile range with the median (black horizontal line); whiskers extend to 1.5× IQR; individual points beyond the whiskers are plotted as outliers. (**b**) Summary statistics (group means ± SEM) of risk scale scores from the automated CSA at the Tokyo Testing Center for PC and NC using biobank-derived urine-based comparison/reference materials (solid fill) and synthetic volatile organic compound reference standards (hatched fill) (*N* = 612 per group). Because only aggregate (summary) statistics were retained for the CSA QC data, panel (**b**) presents group means ± SEM rather than a per-sample distribution; group means and separation metrics (Δ, Cohen’s *d*) are inset within each panel. Both workflows show clear, consistent PC versus NC separation; the CI and risk scale share a common origin—the risk scale is a linear, monotonic transformation of the CI applied for patient-facing interpretability—so although their raw numeric values differ, transformation-invariant metrics (Cohen’s *d*, rank-based separation) are directly comparable (Table 1, Table 2, Table 3 and Table 4).

Over the nine consecutive months of routine clinical operation at the Fukuoka R&D Center, 551 paired PC and NC assays were analyzed manually by trained technicians at the two standard urine dilutions (10^−1^ and 10^−2^), with the actual sample volume processed by the manual workflow during the observation window. Descriptive statistics are summarized in Table 1 and Figure 2. As expected, PC distributions were centered at positive CI values (mean = +0.048 at 10^−1^ and +0.051 at 10^−2^), indicating net attraction of the nematodes toward the PC reference material, whereas NC distributions were centered at negative values (mean = −0.047 and −0.052, respectively), indicating mild repulsion from the NC reference material. The two control groups were thus clearly displaced on opposite sides of CI = 0, the no-preference reference, throughout the entire nine-month period.

**Table 1:** Descriptive statistics for chemotaxis index (CI) values from manual analysis at the Fukuoka R&D Center (January–September 2023, *N* = 551 per group).

Group	*N*	Mean CI	SD	SEM	Median CI	[Min, Max]
PC (10^−1^)	551	+0.048	0.077	0.003	+0.056	[−0.270, +0.266]
NC (10^−1^)	551	−0.047	0.083	0.004	−0.042	[−0.299, +0.185]
PC (10^−2^)	551	+0.051	0.076	0.003	+0.061	[−0.210, +0.233]
NC (10^−2^)	551	−0.052	0.080	0.003	−0.048	[−0.368, +0.163]

Group comparisons confirmed that the PC and NC distributions are highly significantly separated at both dilutions, with large effect sizes (Table 2 and Figure 2). Welch’s *t* = 19.83 (*p* = 5.0 × 10^−75^) at 10^−1^ and *t* = 21.95 (*p* = 8.2 × 10^−89^) at 10^−2^; Cohen’s *d* = 1.19 and 1.32, respectively—both well above the conventional “large effect” threshold (*d* ≥ 0.8). Non-parametric Mann-Whitney U tests yielded identical conclusions (*p* < 10^−68^ in both cases), confirming that the result is not an artifact of distributional assumptions. Levene’s test indicated no statistically significant difference in PC versus NC variances (*p* = 0.09 and *p* = 0.47), supporting the use of a common-variance interpretation of separation.

**Table 2:** Statistical comparison of PC versus NC chemotaxis index distributions (manual analysis, Fukuoka R&D Center; *N* = 551 per group at each dilution).

Dilution	Δ (PC–NC)	Welch’s *t*	*p*-Value (Welch)	Cohen’s *d*	*p*-Value (M-W U)	Levene *p*
10^−1^	+0.096	19.83	5.05 × 10^−75^	1.19	3.26 × 10^−68^	0.09
10^−2^	+0.103	21.95	8.20 × 10^−89^	1.32	7.37 × 10^−80^	0.47

### 3.2. Fully Automated CSA Analysis Yields the Same Discrimination, Run Side-by-Side over the Same Nine Months

In parallel, over the same nine-month period, the fully automated CSA at the Tokyo Testing Center operated continuously under routine clinical laboratory conditions, processing 2448 quality control samples (612 per control type) in addition to all clinical specimens (Table 3 and Figure 2a,b). On the proprietary risk scale used for clinical reporting, the biobank-derived urine-based comparison/reference materials yielded mean risk scale scores of 59.90 (PC) and 45.56 (NC), with a between-group separation of Δ_P–*N* = 14.47 (SEM 0.37 and 0.44, respectively). Synthetic VOC reference standards yielded comparable performance, with means of 59.77 (PC) and 49.59 (NC), and Δ_P–*N* = 10.17 (SEM = 0.51 and 0.52). The somewhat smaller separation observed with synthetic VOC standards is consistent with the simpler chemical composition of synthetic VOC mixtures compared with the full metabolic complexity of authentic urine. The risk scale is a linear, monotonic transformation of the chemotaxis index—purely post-analytical rescaling was applied so that the biological readout can be communicated to patients and clinicians in an interpretable, actionable form—and thus, it re-expresses, rather than re-computes, the discriminative information contained in the CI. A direct statistical consequence follows: a strictly monotonic linear transformation leaves both the standardized effect size (Cohen’s *d*) and rank-based discrimination metrics (Mann-Whitney U, Area Under the Curve (AUC)/probability of superiority) unchanged, so the PC-versus-NC separation quantified on the risk scale is directly and legitimately comparable to that quantified on the raw CI. On this common, dimensionless footing, the two workflows are concordant (Table 4): the manual assay yields Cohen’s *d* = 1.19 and 1.32 at the two dilutions, while the automated CSA yields *d* = 1.44 with biobank-derived urine-based materials and *d* = 0.80 with synthetic VOC standards—all within or above the conventional large effect band (*d* ≥ 0.8). For the most directly comparable, like-for-like contrast (biobank-derived urine-based material processed by both workflows), the standardized separation achieved by the automated platform (*d* = 1.44) meets or exceeds that of the manual workflow.

**Table 3:** Quality control performance of the CSA at the Tokyo Testing Center (January–September 2023; total *N* = 2448; 612 per control type) on the proprietary clinical reporting risk scale.

Reference Standard	Biobank-Derived Urine-Based			Synthetic (VOC Mimic)		
Metric	PC	NC	Δ_P–*N*	PC	NC	Δ_P–*N*
Avg. Risk Scale	59.90	45.56	14.47	59.77	49.59	10.17
SEM	0.37	0.44	0.58	0.51	0.52	0.70

**Table 4:** Scale-independent (standardized) separation of positive-control (PC) from negative-control (NC) distributions in each workflow. Because the CSA risk scale is a linear, monotonic transformation of the chemotaxis index, the standardized effect size (Cohen’s *d*) and the rank-based probability of superiority are invariant to the transformation and are therefore directly comparable across the two readouts. Manual values are computed from the raw CI data (*N* = 551 per group); CSA values are derived from the reported risk scale group means and SEMs (*N* = 612 per group). Probability of superiority is the probability that a randomly chosen PC sample scores higher than a randomly chosen NC sample.

Workflow (Readout)	Reference Standard	Δ (PC–NC)	Cohen’s *d*	*p* (Superiority)
Manual (CI)	Reference material, 10^−1^	+0.096	1.19	0.80
Manual (CI)	Reference material, 10^−2^	+0.103	1.32	0.83
CSA (risk scale)	Biobank-derived urine	14.47	1.44	0.85
CSA (risk scale)	Synthetic (VOC mimic)	10.17	0.80	0.71

### 3.3. The CSA Faithfully Reproduces the Manual Inspection Workflow Step-for-Step

A central design principle of the CSA is replication, not reinvention, of the established manual workflow. Figure 1 maps each step of the manual inspection (handling of nematode recovery, dispensing, chemotaxis incubation, and compute counting) to its automated counterpart on the CSA. The reagents, plate geometry, incubation conditions (30 min at 23 °C), and CI calculation are identical across the two workflows; only upstream sample handling and downstream image-based counting are mechanized. Photographs taken in situ at the Tokyo Testing Center document that the CSA is operated in the actual clinical laboratory environment where N-NOSE testing is conducted, not in a research-only configuration.

## 4. Discussion

The central finding of this study is unambiguous: across nine months of side-by-side, real-world operation, the manual chemotaxis assay performed by trained technicians and the fully automated CSA analysis produced analytically equivalent separation of positive from negative reference controls in the N-NOSE workflow. At the raw chemotaxis index level, the immediate biological readout of the assay, the manual workflow yielded a highly significant, large-effect-size PC-versus-NC separation, and the CSA delivered a comparably robust separation on its proprietary clinical reporting risk scale using both biobank-derived urine-based materials and synthetic VOC reference standards. Because the risk scale is a linear, monotonic transformation of the CI, standardized separation is directly comparable across the two readouts and is concordant, so the manual and automated processes show no meaningful difference in discriminative performance. Two datasets, acquired independently at two operational sites under their actual routine clinical laboratory conditions, converge on the same conclusion through methodologically distinct observations.

This equivalence is to be expected on scientific grounds: the CSA automates only the preparatory and readout steps surrounding the biological detection event. The nematode itself, which performs the actual cancer-associated VOC sensing through its native olfactory receptor repertoire [1,2,3,4,5], remains the unchanged analytical element in both workflows. This situation differs fundamentally from mechanization domains in which automation can alter the detection principle or product quality (for example, fully automated food manufacturing, where ingredients, processing, and final-product chemistry all change relative to the artisanal version). In the N-NOSE context, the biological transducer is decoupled from the sample-handling apparatus; the CSA simply provides a more standardized, higher-throughput interface for delivering identical samples to and reading identical behaviors from the same biological sensor. Analytical equivalence is therefore the predicted, unsurprising outcome, and our data confirm that prediction at a statistically conclusive level (Table 1, Table 2, Table 3 and Figure 2).

Several methodological considerations merit comment. First, the CSA risk scale score is a linear, monotonic transformation of the raw CI, applied post-analytically only to render the biological readout interpretable and actionable for patients and clinicians. Because a strictly monotonic linear transformation leaves standardized effect sizes and rank-based discrimination metrics unchanged, the CI-level and risk scale analyses quantify the same underlying separation expressed on two different scales; presenting both, and confirming their concordance through transformation-invariant effect sizes (Table 4), places the between-workflow comparison on a common, dimensionless footing rather than confounding it with post-analytical presentation differences. Second, the use of both biobank-derived urine-based comparison materials and synthetic VOC reference standards in the CSA QC program provides dual-modality validation: biobank-derived urine-based materials confirm performance with authentic biological matrices, while synthetic VOC standards offer the batch-to-batch chemical reproducibility advantages required for long-term QC monitoring. Third, while a fully paired, same-sample, same-day cross-site comparison would represent the ideal experimental design and is a natural next step, the present longitudinal QC approach has the pragmatic advantage of reflecting actual real-world operating conditions and the actual sample volumes processed by each workflow. Accordingly, the present design establishes two things: that each workflow independently achieves robust, stable PC-versus-NC discrimination under genuine operating conditions and—through transformation-invariant effect sizes—that the magnitude of that discrimination is concordant between workflows. It remains a longitudinal, real-world QC comparison rather than a formal same-sample, same-day paired method comparison (e.g., a CLSI EP09-style regression and Bland-Altman analysis on identical specimens); such a paired study on the automated platform is the definitive confirmatory step and is planned. We therefore frame our conclusion as concordant discriminative performance at the level of QC, not as numeric interchangeability of the two readouts.

These findings have several implications. For regulatory purposes, the demonstrated equivalence provides peer-reviewed evidence that automating the N-NOSE inspection process does not compromise analytical reliability, a foundational prerequisite for discussions with agencies such as the U.S. FDA regarding test validation and potential market authorization. Generally, the approval process for such in vitro diagnostic devices does not require “blinded” trials, due to the lack of any human-related bias in the test results. N-NOSE uses living organisms (nematodes) for cancer screening tests; however, the technician’s intentions do not influence the nematode behavior or the data acquisition process (image capture and following automatic CI calculation). This study also demonstrates that even within the workflow at the N-NOSE operation facility (testing center), where the entire process of nematode chemotactic behavior analysis is fully automated, the test exhibits equivalent performance and, like other in vitro diagnostic devices, shows no human-related bias in the test results. These same properties make large-scale, double-blinded validation practical rather than problematic. Because the CSA removes operator involvement from sample handling and performs worm counting and CI computation by machine vision, control or clinical status can be fully masked (for example, by barcoding), with no possibility of operator influence on the nematodes or the readout, and the substantial routine testing volume already processed in Japan [20] confirms the throughput headroom that such studies require. For the broader research community, these data provide a real-world reference documenting the CSA’s performance characteristics and will support future large-scale clinical validation studies conducted on the automated platform [14,15,16,17,20]. Independent academic groups in the United States and Europe have already demonstrated, with manual assays, that the *C. elegans* chemotaxis principle reliably discriminates cancer from non-cancer urine [7,18]; by showing that automation preserves that principle, the present work positions the CSA as a fit-for-purpose platform for scaled clinical and translational research. Finally, the findings reinforce confidence in the existing body of N-NOSE clinical evidence and complement prior reviews of the platform [6,9,10,11,12,13,14,15,16,17,20,22], many of which were generated under the same QC framework described here.

## 5. Conclusions

This nine-month real-world, side-by-side study demonstrates that manual chemotaxis analysis performed by trained technicians and fully automated analysis on the Chemotaxis Scoring Apparatus (CSA), each operated under genuine routine clinical laboratory conditions, produce large, statistically equivalent separation of positive from negative reference controls in the N-NOSE workflow. At the raw chemotaxis index level, the manual assay delivers reproducible discrimination over the full nine-month period, and the CSA delivers comparably robust discrimination on its proprietary clinical reporting risk scale using both biobank-derived urine-based materials and synthetic VOC reference standards. Because the risk scale is a linear, monotonic transformation of the CI, the standardized separation is directly comparable across the two readouts and is concordant, indicating no meaningful difference in discriminative performance between the manual and automated processes. Because the CSA replicates rather than replaces the biological detection event, this equivalence is the predicted outcome, and our data confirm it under operational conditions, at scale. These results validate the analytical reliability, reproducibility, and real-world consistency of the automated N-NOSE testing platform and provide a peer-reviewed foundation for forthcoming regulatory submissions and large-scale clinical validation studies using the CSA.

## Data Availability

The data presented in this study are available upon request from the corresponding author due to restrictions on sharing proprietary internal R&D data.

## Abbreviations

The following abbreviations are used in this manuscript: AUC, area under the (receiver operating characteristic) curve; CI, chemotaxis index; CLSI, Clinical and Laboratory Standards Institute; CSA, Chemotaxis Scoring Apparatus; FDA, U.S. Food and Drug Administration; GPCR, G-protein-coupled receptor; M-W U, Mann-Whitney U; MCED, multi-cancer early detection; N-NOSE, Nematode-NOSE; NC, negative control; NGM, nematode growth medium; PC, positive control; QC, quality control; SD, standard deviation; SEM, standard error of the mean; VOC, volatile organic compound.
